# Hemophagocytic lymphohistiocytosis and visceral leishmaniasis in children: a series of cases and literature review

**DOI:** 10.1590/1984-0462/2022/40/2020269

**Published:** 2021-09-01

**Authors:** Natália Fernanda Ferreira Brum, Julia Sampaio Coelho, Laís Silva Carvalho, Matheus Nascimento Otoni Vieira, Aline Almeida Bentes, Ericka Viana Machado Carellos, Lilian Martins Oliveira Diniz, Andrea Lucchesi de Carvalho, Roberta Maia de Castro Romanelli

**Affiliations:** a Universidade José do Rosário Vellano, Belo Horizonte, MG, Brazil.; b Universidade Federal de Minas Gerais, Belo Horizonte, MG, Brazil.; c Hospital Infantil João Paulo II, Fundação Hospitalar do Estado de Minas Gerais, Belo Horizonte, MG, Brazil.

**Keywords:** Leishmaniasis, visceral, Lymphohistiocytosis, hemophagocytic, Diagnosis, Drug therapy, Leishmaniose visceral, Linfo-histiocitose hemofagocítica, Diagnóstico, Tratamento farmacológico

## Abstract

**Objective:**

Hemophagocytic lymphohistiocytosis syndrome (HLHS) is characterized by an immunological hyperactivation of cytotoxic T cells, natural killer cells, and macrophages, leading to the secretion of proinflammatory cytokines. HLHS associated with Visceral Leishmaniasis might be difficult to diagnose once symptoms are similar, resulting in the death of untreated patients. Our aim is to describe a series of cases of Visceral Leishmaniasis with HLHS admitted to a referral hospital for infectious diseases.

**Case description:**

All 115 cases of Visceral Leishmaniasis referred to a referral center for pediatric infectious diseases were reviewed to identify the cases of HLHS. Five cases (4.5%) were confirmed with HLHS and they presented fever, splenomegaly, cytopenia, hypertriglyceridemia or hypofibrinogenemia, increased ferritin and hemophagocytosis in the bone marrow.

**Comments:**

It important to rule out HLHS in children with infectious diseases that do not respond adequately to treatment or in patients with severe symptoms, especially in leishmaniasis endemic areas.%

## RESUMO

**Objetivo:**

Linfo-histiocitose hemofagocítica (LHH) é uma síndrome caracterizada pela hiperativação de linfócitos T citotóxicos, células *natural killer* e macrófagos, levando à secreção de citocinas pró-inflamatórias. Linfo-histiocitose hemofagocítica associada a Leishmaniose Visceral é um quadro de grande desafio para o diagnóstico, considerando que os sintomas são semelhantes, podendo levar ao óbito pacientes não tratados. Nosso objetivo é descrever uma série de casos de Leishmaniose Visceral com LHH admitidos em um hospital de referência para doenças infecciosas.

**Descrição do caso:**

Todos os 115 casos de Leishmaniose Visceral encaminhados ao centro de referência em doenças infecciosas pediátricas foram revistos para identificar os casos de LHH. Cinco casos (4,5%) foram confirmados com LHH e apresentaram febre, esplenomegalia, citopenia, hipertrigliceridemia ou hipofibrinogenemia, aumento de ferritina e hemafagocitose em medula óssea.

**Comentários:**

Deve-se suspeitar de LHH em crianças com doenças infecciosas que não respondem adequadamente ao tratamento ou em pacientes com sintomas graves, principalmente em áreas endêmicas da leishmaniose.%

## INTRODUCTION

Hemophagocytic lymphohistiocytosis syndrome (HLHS) is a disorder characterized by an immune hyperactivation of cytotoxic T cells, natural killer (NK) cells, and macrophages, leading to the secretion of high levels of proinflammatory cytokines.^1^ ^,^ ^2^ The primary form is an inherited disorder with autosomal recessive transmission.^3^ The secondary form occurs after immunological dysregulation induced by neoplasia, autoimmune diseases, or systemic infections.^4^ Epstein-Barr virus is the most frequently reported infectious cause.^5^

Visceral leishmaniasis (VL) is a systemic disease that affects the reticuloendothelial system, caused by *Leishmania*, transmitted through bites of infected sandflies. HLHS associated with VL might be difficult to diagnose once symptoms are similar, resulting in death if left untreated.^6^ ^,^ ^7^

The diagnostic criteria for HLHS are based on clinical, laboratory, and morphological data and must include 5 of the following 8 criteria:^8^ 1) fever; 2) splenomegaly; 3) cytopenia affecting 2 or more peripheral blood series (hemoglobin <9g/dL, thrombocytopenia <100,000/mm^3^, neutrophils <1,000/mm^3^); 4) hypertriglyceridemia (≥265mg/dL) and/or hypofibrinogenemia (≤1.5g/L); 5) hemophagocytosis in the bone marrow, spleen, or lymph nodes; 6) low or absent NK cell activity; 7) hyperferritinemia (ferritin ≥500μ/L); and 8) elevation of CD25 ≥2,400U/mL.

The treatment of primary HLHS consists of immunosuppressive drugs. However, in cases associated to VL, patients should be treated with liposomal amphotericin (LA) and, only if they do not respond well to specific treatment, should immune modulation be considered.^8^ ^–^ ^11^

It should be emphasized that in an endemic area for VL such as Belo Horizonte,^12^ HLHS should be considered in these patients. A series of cases of VL with HLHS admitted to an infectious diseases referral hospital were describe here.

## CASE REPORTS

This was a descriptive study performed in Belo Horizonte, Minas Gerais, Brazil, from January 2016 to April 2018. All cases of VL referred to pediatric infectious diseases center were reviewed and cases of HLHS were identified according to diagnostic criteria.^8^ The study was approved by the Ethics Committee of *Fundação Hospitalar do Estado de Minas Gerais* (FHEMIG – CAAE number: 33547520.7.0000.5119; report number 4.127.269)

A total of 115 cases of VL were assisted at the referral center and five cases of HLHS were described. Clinical manifestations are presented below and clinical and laboratory criteria for HLHS are described in Table 1.

**Table 1:** Clinical and laboratory parameters found in five cases of hemophagocytic syndrome associated with Leishmaniasis.

		Case 1	Case 2	Case 3	Case 4	Case 5
Fever		+	+	+	+	+
Splenomegaly		+	+	+	+	+
Cytopenias (2 or more)		Hb: 6.9		Hb: 5.6	Hb: 6.1	
	Hb (g/dL)	Plq: 49,000	Hb: 6.0	Hb: 7.2	Plq: 49,000	Hb: 7.8
	Plq cels/mm^3^	Hb: 7.2	Plq: 88,000	Hb: 7.2	Neu: 750	Neu: 875
	Neu (cels/mmm^3^)	Plq: 51,000				
Hypertriglyceridemia (mg/dL) or Hypofibrinogenemia (g/L)		TG: 680	TG: 416 F: 102	TG: 300	TG: 407	TG: 264* F<150
Serum ferritin (μ/L)		>1,000	1,875	>1,000	>1,000	>1,000
Hemophagocytosis in bone marrow		+	+	+	+	+
Reduction of NK7 activity		ND	ND	ND	ND	ND
CD25 increased		ND	ND	ND	ND	ND

Hb: hemoglobin; Neu: neutrophils; Plq: platelets; TG: triglycerides; F: fibrinogen; ND: not done;

* borderline value for hypertriglyceridemia considering the criteria established by the International Society of Histiocytosis (2004).^8^

### Case 1

A 14-month-old female infant was admitted with fever, hepatosplenomegaly, bicytopenia, hypoxemia, and increased serum ferritin. A myelogram revealed *Leishmania* spp. and hemophagocytosis. She presented liver failure, and lipossomal amphotericin B and corticosteroid were started. She was treated with cefepime and vancomycin due to sepsis (blood cultures revealed *Klebsiella* spp., and she had skin lesions). After 12 days, she was discharged from the hospital and continued oral corticosteroid therapy.

### Case 2

A 7-month-old male infant presented with fever for 10 days associated with hepatosplenomegaly, bicytopenia, pallor, anasarca, and increased ferritin. Immunochromatographic rapid test with rK39 antigen was positive for *Leishmania* spp., and amastigotes were detected in a myelogram, which also presented hemophagocytosis. Complete treatment with lipossomal amphotericin was started and cefepime was used due to fever-associated neutropenia. In addition, he underwent transfusion of platelets, red blood cells, and albumin. After a diagnosis of HLHS, corticotherapy was started. The patient recovered after 3 days of treatment.

### Case 3

A one-year-old male child was admitted with a history of continuous fever for 3 months and hepatosplenomegaly, anemia, hypertriglyceridemia, increased ferritin, and hemodynamic instability, requiring blood transfusion. Immunochromatographic rapid test with rK39 antigen was positive for *Leishmania* and the myelogram presented *Leishmania* spp with hemophagocytosis. Then, glucantime was started. As fever persisted, on day 8, nonresponse to glucantime was considered and it was replaced by lipossomal amphotericin. Due to the fever and neutropenia, cefepime was also started. After further testing, the patient fulfilled 5 of the 8 HLHS criteria. After clinical and laboratory improvement, cefepime was suspended on day 9 and the patient was discharged on day 12.

### Case 4

A female infant aged 2 years was admitted presenting with fever for 4 days, oliguria, generalized edema, and hematochezia for 2 days. She was pale, with hepatosplenomegaly and anasarca. Laboratory tests revealed pancytopenia. Cefepime was started due to neutropenia and clinical instability, A myelogram revealed *Leishmania* spp. and hemophagocytosis. On day 2, lipossomal amphotericin was started and a blood transfusion was necessary. On day 3, HLHS was confirmed. However, on day 5, she presented clinical worsening, with respiratory insufficiency, hypoalbuminemia, and a corticosteroid was initiated. On day 8, the patient improved, with resolution of fever and neutropenia and a negative blood culture resulted in discontinuation of the antimicrobial. She was discharged on day 15 and continued oral corticosteroid therapy for 8 weeks.

### Case 5

A 9-year-old male child was admitted to the hospital with fever and headache for 12 days associated with polyarthralgia. He had autoimmune hepatitis treated with prednisone, azathioprine, and vitamin D. He was pale with hepatosplenomegaly and jaundice. On day 2, the patient was diagnosed with VL by means of an immunochromatographic rapid test with rK39 antigen and hemophagocytic syndrome and lipossomal amphotericin was started. The child also presented increased ferritin, hypertriglyceridemia, and hypofibrinogenemia. Because of the febrile neutropenia in an immunosuppressed patient, cefepime was started on day 5. On day 8, lipossomal amphotericin was completed. Due to clinical and laboratory improvement, the antibiotic was finished and the child was discharged on day 11.

## DISCUSSION

In this 2-year survey at a referral center for infectious diseases, 4.4% of patients with lipossomal amphotericin were suspected to have HLHS. Of these, only one did not meet the criteria needed for the diagnosis owing to a borderline triglyceride level (Table 1). In a retrospective study conducted at a referral center in the same city, 7 children had a confirmed HLHS diagnosis, 2 of which were associated with lipossomal amphotericin. Hemophagocytosis was detected in the bone marrow tissue of 6 patients.^2^

HLHS is a syndrome of immune hyperactivation and release of cytokines, characterized by clinical signs and symptoms of severe and uncontrolled inflammation. Although rare in adults, it is frequently seen in the pediatric population caused by several infectious diseases.^13^

A 5-year review of the literature revealed only 32 case reports of HLHS, with most cases being secondary to Epstein-Barr virus infection.^14^ A 1-year multicenter prospective study in Spain, identified 24 children with VL and 10 presented with associated HLHS.^1^ In the present series, a higher frequency of cases was found related to leishmaniasis in the pediatric population in a shorter period of time. All children presented six criteria (fever; splenomegaly; cytopenia; hypertriglyceridemia or hypofibrinogenemia, hyperferritinemia, and hemophagocytosis) in the bone marrow. Only NK cell activity and CD25 are not routinely performed in the service.

VL should be ruled out in all children with HLHS, as the symptoms of the infection may overlap with those of the HLH syndrome and could result in death if left untreated.^15^ VL should be considered especially for children in endemic areas.^4^

Although the treatment of HLHS includes immunosuppressive drugs, it can be resolved with specific treatment of leishmaniasis.^16^ In a study in Spain,^17^ 8 children with leishmaniasis and HLHS had improvement with specific treatment of only LA or sodium stibogluconate. Scalzone et al.^4^ reported a case of HLHS in a 7-month-old infant undergoing immunosuppressive therapy who improved only when a second myelogram showed *Leishmania* spp. 3 months later and lipossomal amphotericin was started. Domínguez-Pinilla et al.^10^ and Higel et al.^11^ reported similar cases and suggest that patients with VL-associated HLHS should be promptly treated with lipossomal amphotericin, considering that they do not respond well to immune modulation.

In the present series, all patients received lipossomal amphotericin, and only 1 patient did not receive corticosteroid therapy. However, 2 patients had severe disease with hemodynamic instability and another patient used corticosteroids due to autoimmune hepatitis. Immunosuppressive drugs should be reserved for patients who do not respond within the first 2 to 3 days of specific treatment.^18^ Lipossomal amphotericin is currently considered the first-line treatment for VL, with lower toxicity and acceptable efficacy.^19^

Both HLHS and VL are often associated with neutropenia, inducing infections and antibiotics are used during febrile neutropenia,^18^ ^,^ ^20^ as observed in this study.

This report reinforces the importance of suspecting HLHS in children with infectious diseases that do not respond adequately to treatment or with severe symptoms, especially in leishmaniasis endemic areas.
